# Grand Challenges in Global Health: Community Engagement in Research in Developing Countries

**DOI:** 10.1371/journal.pmed.0040273

**Published:** 2007-09-11

**Authors:** Paulina O Tindana, Jerome A Singh, C. Shawn Tracy, Ross E. G Upshur, Abdallah S Daar, Peter A Singer, Janet Frohlich, James V Lavery

## Abstract

The authors argue that there have been few systematic attempts to determine the effectiveness of community engagement in research.

The Bill & Melinda Gates Foundation (http://www.grandchallengesgh.org), the United States National Institutes of Health (http://grants1.nih.gov/grants/index.cfm), the United Kingdom Wellcome Trust (http://www.wellcome.ac.uk/funding), and others are increasing funding for research on diseases that affect the world's poor. The goal is to develop superior diagnostic tools, prevention strategies, and interventions to counter the debilitating impact of these diseases. Successful completion of this research and adoption of the resulting technologies will depend on successful engagement with the intended beneficiaries. Recent research in developing countries, such as the abandoned trials in Cameroon and Cambodia of tenofovir as pre-exposure prophylaxis against HIV infection [1], has shown that even in studies where ethical issues have been addressed, challenges related to community engagement (CE) can still undermine the research.

Various CE models exist in the fields of public health, community planning [2], governance, and community development. However, there have been few systematic attempts to determine the effectiveness of CE in research. As an advisory service on ethical, social, and cultural (ESC) issues for the Grand Challenges in Global Health (GCGH) initiative, discussed in the first article in this series [3], we are exploring a range of ESC issues identified by the GCGH investigators and developing world key informants, discussed in the second article in this series [4]. The investigators and key informants placed particular emphasis upon the importance of community engagement, and therefore we prepared a conceptual paper on this topic, which we distributed as a working paper to GCGH investigators and program staff at the 2nd Annual GCGH Meeting. In this article, we summarize this conceptual paper. We first examine the concept of CE in research in developing countries, then we describe published models of CE, and finally we discuss two relevant examples of CE in research from Africa.

## What Is a Community?

There is no standard definition of a community. The term “community” has been used to describe interactions among people in primarily geographic terms [5]. But it is now accepted that people who live in close proximity to one another do not necessarily constitute a community, since they may differ with respect to value systems and other cultural characteristics that are more relevant to the social concept of community.

Some have argued that the defining feature of a community is the common identity shared by its members [6]. Thus, a single individual may belong simultaneously to different religious, vocational, or ethnic communities, or communities with distinct values and aspirations may inhabit a single geographic area. Even though community is determined largely by shared traditions and values, communities are not static and may accommodate multiple and even conflicting interpretations of their own traditions and values [7]. Outsiders may also define community differently from insiders.

Charles Weijer and colleagues [8] provide a comprehensive account of features of community in relation to research (Box 1). The extent to which a community reflects these features is a measure of its cohesiveness. The authors argue that different levels of community cohesiveness or specific features may warrant different research protections. Such protections might include consultation in protocol development, information disclosure about proposed research and informed consent, involvement in research conduct, access to data and collected samples, and involvement in the dissemination and publication of the research results [9]. Brunger and Weijer have argued, in the context of a study of ethnobotany and indigenous knowledge, that the community constitutes the collection of individual people who share research-related risks [10].

**Box 1. Features of a Community in Relation to Research**

- Common culture and traditions, cannon of knowledge, and history
- Comprehensiveness of culture
- Health-related common culture
- Legitimate political authority
- Representative group/individuals
- Mechanism for priority setting in health care
- Geographic localization
- Common economy/shared resources
- Communication network
- Self-identification as community (Based on [8,9])

## What Is Community Engagement?

Given the complexity of the concept, it is not surprising that there is no universally accepted definition of CE (Table 1). In our view, the concept of engagement in research goes beyond community participation; it is the process of working collaboratively with relevant partners who share common goals and interests. This involves “building authentic partnerships, including mutual respect and active, inclusive participation; power sharing and equity; mutual benefit or finding the ‘win-win’ possibility” [11] in the collaborative initiative.

**Table 1:**
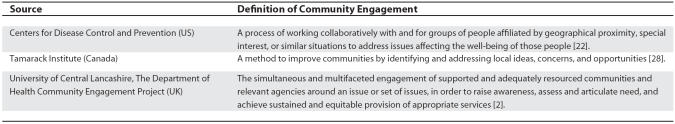
Example Definitions of Community Engagement in Research

The terms “community participation” and “community involvement” both connote manifestations of CE, particularly in the social science literature, and have been influential in CE approaches. For example, the HIV Prevention Trials Network has developed a “toolbox” for community participation in HIV trials [12]. The toolbox aims to encourage collaborative and participatory efforts by both researchers and members of the community to ensure that the research activities are responsive to the needs of the host community.

Another example of community engagement in research is “community consultation,” a goal that can be satisfied by the establishment of community advisory boards. Quinn has argued that such boards “provide a mechanism for community consultation that contributes to protecting communities and fostering meaningful research” [13].

“Collaborative partnership” is another way of framing CE. In the context of international research, such partnership has been proposed as an ethical requirement [14]. CE involves the need for researchers to develop partnerships with local stakeholders and to involve them in assessing local health problems, determining the value of research, planning, conducting and overseeing research, and integrating research into the health care system [15].

## Goals of Community Engagement in Research

The idea of CE as an ethical requirement for research involving human participants, particularly marginalized populations, has made its way into international research ethics guidelines and reports from organizations such as the Council for International Organizations of Medical Sciences [16], the US National Bioethics Advisory Commission [17], and the UK's Nuffield Council on Bioethics [18]. CE activities represent efforts to ensure authentic and appropriate authorization and permission for research undertaken within specific communities, with appropriate levels of community involvement in, and ownership of, these activities. Several specific goals for CE are listed in Box 2. At a more general level, Dickert et al. have identified four ethical goals—enhancing protection, enhancing benefits, creating legitimacy, and sharing responsibility—that are facilitated through the incorporation of a community's views and its participation in research [19].

With increasing research in developing countries, these CE goals have become prominent in research policy. Such research activities have had a poor record to date of actually benefiting host communities, and there is growing recognition that communities, not just individuals, can suffer harm from participation in research [20]. For example, without adequate protections, population genetics research runs the risk of stigmatizing or discriminating against recognizable communities, while environmental health research can end up exposing poor and otherwise marginalized communities to an unfair burden of research risks [8, 29]. CE is increasingly viewed as a meaningful response to these problems.

## Conceptual Models of Community Engagement

Various CE models have been proposed, especially in research involving Aboriginal communities. The Canadian Institutes of Health Research “Guidelines on Health Research Involving Aboriginal People” [20] recommend a participatory research approach, in which community members are active participants at every stage of the research process. These guidelines and others recommend the inclusion of cultural knowledge in research under mutually agreed terms, and with the guidance of the knowledge holders in the community.

The Effective Interventions Unit of Scotland [21] has proposed 16 guiding principles for CE under three major headings: planning, commitment, and inclusiveness. Although this conceptual model was developed in the context of tackling drug-related issues primarily in urban centers in Scotland, the principles are relevant to health research activities in developing countries. The US Centers for Disease Control and Prevention have also recommended nine guiding principles for engaging communities in research [22]. This framework recommends flexibility in engagement efforts to meet the changing needs of the community. Another basis for a CE conceptual model more specific to research in developing countries comes from the benchmarks for ethical research of Emanuel et al. [14]. Although these benchmarks are organized around the idea of collaborative research partnerships, rather than the broader concept of CE, their general thrust is similar to the other models. Table 2 provides links to CE conceptual models and their recommended principles.

**Table 2:**
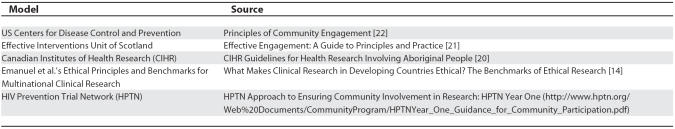
Conceptual Models of Community Engagement and Their Principles

We have selected these examples from the literature to illustrate three key points. First, the general aims of these models are similar, although some goals, such as making research ethical or improving health promotion, are specific to individual models. Second, the contexts for which the models have been developed clearly influence the nature of the models. For example, the Canadian Institutes of Health Research guidelines place greater emphasis on respect for community traditions and ownership than the model developed for combating urban drug addiction. Third, although they share similarities, these models also illustrate some of the diversity in thinking about CE which, in turn, highlights the complexity of evaluating the effectiveness of CE in research.

## Community Engagement in Practice

The principles and recommendations outlined above provide tools for promoting partnerships between investigators and host communities of research. But precisely how the *effectiveness* of CE should be conceptualized and measured has not been established. In fact, quite aside from the evaluative questions, there are few well-documented examples of CE in practice, especially in international collaborative research.

In this section, we provide a brief description of two relevant case examples of CE: the Centre for the AIDS Program of Research in South Africa (CAPRISA), and the Navrongo Experiment in Ghana.

**Box 2. Goals of Community Engagement**

- To ensure the relevance of research (e.g., National Bioethics Advisory Commission, Nuffield Council on Bioethics) [17,18]
- To assess whether relevant research is culturally and practically acceptable in the context it is intended (e.g., Council for International Organizations of Medical Sciences) [16]
- To ensure that community disruption is minimized, i.e., avoiding the displacement of local medical staff from pressing local needs [26]
- To avoid exploitation, by ensuring a fair distribution of the benefits of research [14]
- To take into account the ethical hazards that may be part of the social, economic, and political landscape of the community [27]

## Enhancing Community Response to Research: The CAPRISA Model

CAPRISA (http://www.caprisa.org/), an AIDS research institute in Durban, South Africa, has developed a CE program with the community of Vulindlela, a rural area about 160 kilometers from Durban. The purpose of the CAPRISA Community Program is to support and facilitate community involvement and informed participation in all CAPRISA projects starting at an early stage of protocol development through to data collection.

Vulindlela, like CAPRISA's other two research sites, has its own community advisory committee. Representatives from each community advisory committee, plus additional members, make up the overarching CAPRISA Community Advisory Board. CAPRISA has also appointed a community liaison person, whose primary role is to consult with relevant nongovernmental organizations, health care workers, community opinion leaders, and study participants on CAPRISA-related research matters. In addition, CAPRISA has established “community research support groups” (CRSGs) which are site- and study-specific bodies aimed at preparing the local community for participation in specific CAPRISA research projects. Projects include the host response to acute infection study (http://www.caprisa.org/Projects/acute_infection.html), sponsored by the US National Institutes of Health, and a tenofovir-based microbicide trial (http://www.caprisa.org/Projects/microbicides.html), sponsored by the US Agency for International Development.

The CRSGs provide community input to CAPRISA investigators on issues such as study participant recruitment and retention strategies, cultural factors that might affect the research initiative, and development of study-specific communication strategies in Zulu, the indigenous language of the area. They form an ongoing dialogue between CAPRISA investigators and the host communities. CAPRISA convenes monthly meetings with the CRSGs, although CAPRISA encourages the CRSGs and/or other members of the community to raise research-related concerns at any time with the CAPRISA Vulindlela site manager responsible for the study clinic.

Research results are fed back to the community at monthly CRSG meetings. These meetings take the form of plenary addresses, although additional focus group discussions are also arranged to discuss particular issues. These focus groups give study-specific cohorts or sub-communities (for example, youth or female participants) the opportunity to raise concerns or ask sensitive questions they would not ordinarily be comfortable enough to raise in the larger plenary sessions, which usually contain a broader community representation.

CAPRISA is currently in the process of empirically measuring the impact of these initiatives on participant recruitment, retention, and positive feedback. Investigators view this CE infrastructure positively and are confident these initiatives have had an empowering effect on the community. Prior to CAPRISA's presence at Vulindlela, the local community had little knowledge of HIV research, and discussion of HIV/AIDS at traditional community gatherings was considered taboo. Open discussion of HIV/AIDS is now common at traditional gatherings, and posters raising awareness of the pandemic are a regular feature. Many community members credit CAPRISA's engagement efforts with sensitizing the community to the HIV/AIDS pandemic, helping to reduce the stigma attached to the disease, and enabling relevant, world-class HIV/AIDS research in the region.

## Introducing Primary Health Care Delivery to Rural Communities: The Navrongo Model

In 1994, the Navrongo Health Research Centre initiated a community-based research project in the Kassena-Nankana District of northern Ghana, to develop, test, and evaluate approaches to rural health service delivery using a combination of strategies (http://www.ghana-chps.org/navrongo.htm). With the support and approval of the Ministry of Health, the Centre embarked on a series of consultations with the chiefs and residents of the district, who contributed to the design of the project, known as the Navrongo Experiment [23].

The consultations with chiefs and residents helped to establish mutual trust between researchers and the community, which has been sustained over the years. The key stakeholders in this project were community leaders, traditionally known as chiefs, district health authorities, development partners, and researchers. The initiative made community leaders local consultants to the project, and involved them at all stages of implementation. The process of consulting local authorities, opinion leaders, and household heads about any new activity in the community, including research, follows a long-established protocol [24] that has become a model for public health interventions in Ghana. This approach has been incorporated into a policy known as the Ghana Community-Based Health Planning and Services Initiative [23], which has been adopted by several districts within the country.

Unique features of the Navrongo model include community entry, a process of going into the community to meet with community leaders before initiating a research activity, and community “durbars.” Durbars involve a gathering of chiefs, elders, opinion leaders, and community members, along with researchers, to deliberate on a proposed research agenda, and to consolidate and communicate community views and concerns. Durbars have been used to mobilize the community for discussions about proposed research projects, and to provide feedback on research activities to the community. The concept of durbars also demonstrates how cultural institutions can be utilized for mobilizing communities and promoting the exchange of ideas.

A publication called *What Works, What Fails* shares the experiences of the Navrongo Experiment. It notes that while community participation is important, translating the concept into practical terms at the local level can be difficult. “Significant institutional, economic, social, health and environmental concerns of community members must be addressed if efforts are to succeed” [23].

## Next Steps

Although the importance of CE in international collaborative research has been recognized and numerous CE models have been described for application in different contexts, there is little empirical data on the effectiveness of CE in international collaborative research. As CE becomes more widely expected as a feature of ethical international collaborative research, it will become important to identify good CE practices and be able to describe in detail how they contribute to CE effectiveness. A recent commentary [25] has called for an empirical approach to CE, so that lessons and insights can be reliably documented and applied in future projects.

One of the main aims of our work is to develop an account of effectiveness in CE in global health research. To do this, we have undertaken a global CE case study in international collaborative research. Using individual case studies from various research projects in developing countries, we will document and analyze community engagement efforts and identify good practices from multiple stakeholder perspectives. From these insights, we hope to develop some preliminary guidelines to facilitate CE for researchers and communities. We hope this work will contribute to improvements in CE in research.

## Abbreviations

CAPRISA: Centre for the AIDS Program of Research in South Africa
CE: community engagement
CRSG: community research support group
ESC: ethical, social, and cultural
GCGH: Grand Challenges in Global Health

## Note Added in Proof

Reference 29 is cited out of order in the article because it was added while the article was in proof.
